# Supplementary material for: The Abundance and Taxonomic Diversity of Filterable Forms of Bacteria during Succession in the Soils of Antarctica (Bunger Hills)
Source: Microorganisms. 2021 Aug 13;9(8):1728. doi: 10.3390/microorganisms9081728 (PMC8400457; doi:10.3390/microorganisms9081728)
Supplement: Supplementary file 1 [file microorganisms-09-01728-s001.zip › microorganisms-1299247-supplementary.pdf]

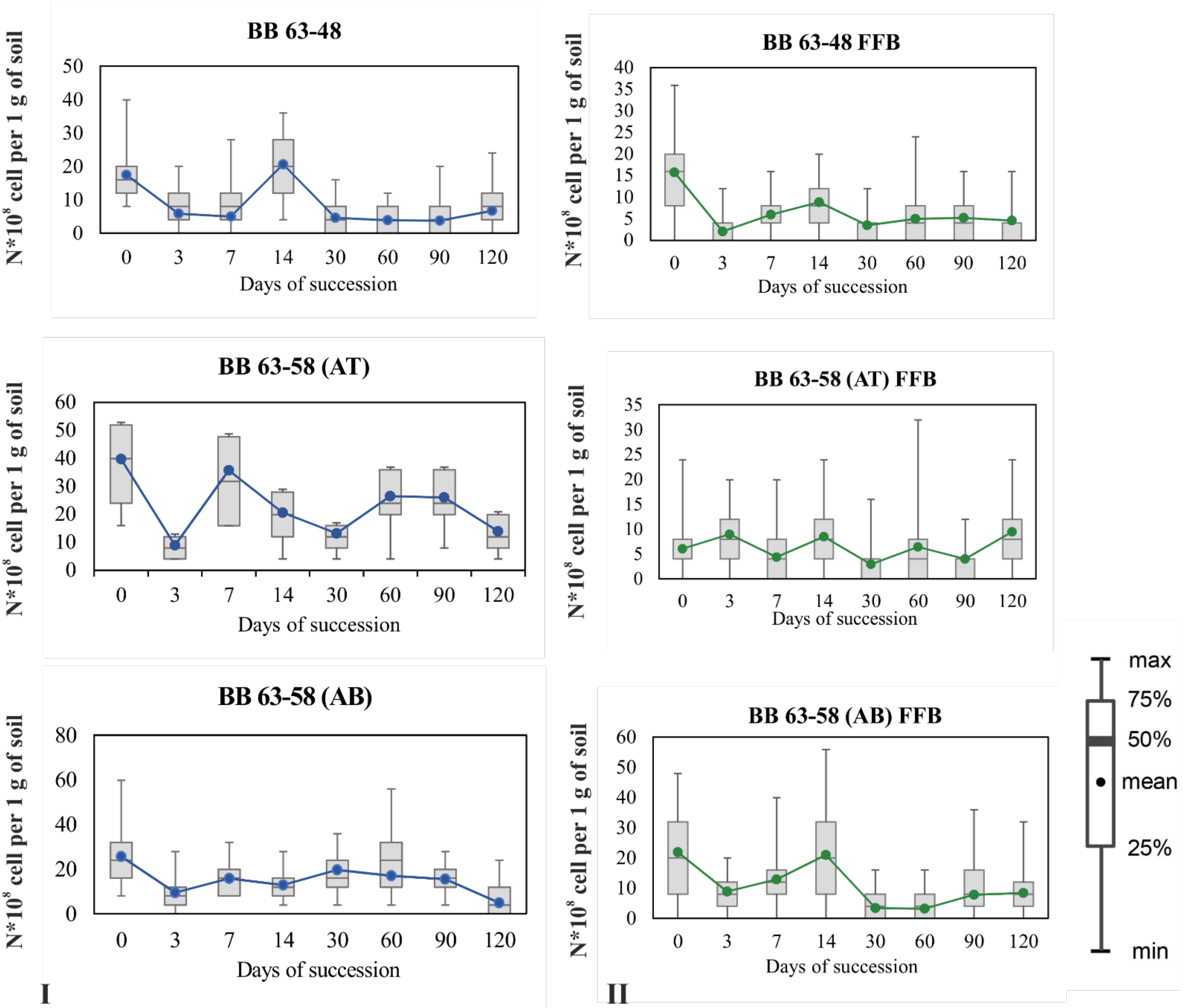

Figure S1. Dynamics of the total number of bacteria and FFB in the studied soils, incubation temperature 5°C: I – soil (total cells); II – FFB

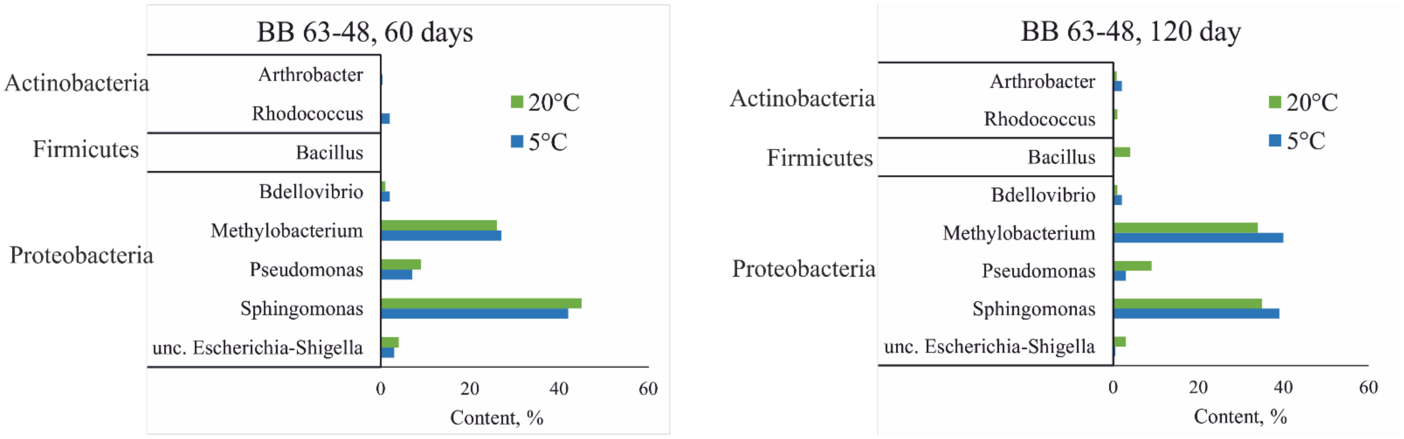

Figure 2. Distribution of dominant phyla in sample BB 63-48

Table S1. OTUs which are present only in the filtrate / in the soil

Phylum	Class	The filtrate	Soil suspension
Proteobacteria	*Alpha-proteobacteria*	*Ochrobactrum*	*Rhodospirillaceae*
		Roseomonas	*Polymorphobacter*
		Paracoccus	Uncultured (*Sphingomonadales*)
		Afipia	Uncultured (*Rickettsiales*)
			Uncultured (Cand. *Odyssella*)
			Uncultured (*Holosporaceae*)
			Uncultured (*Rickettsiaceae*)
			Rhizobiales
			Devosia
			Ambiguous taxa (LWSR-14)
			Rhodoplanes
			Methylocella
			Inquilinus
	Beta-proteobacteria	*Achromobacter*	Uncultured (*Comamonadaceae*)
		Polarmonas	*Limnobacter*
		Delftia	*Nitrosomonadales*
		Ralstonia	Uncultured (*Neisseriales*)
		Methylophilaceae	*Rhodocyclales*
		Hydrogenophilaceae	*Noviherbaspirillum*
			Uncultured *Alcaligenaceae*
	Delta-proteobacteria	-	*Phaselicystis*
			Ambiguous taxa (KD3-10)
			Polyangiaceae (Blrii41)
			Uncultured *Desulfovibrionales*
			Uncultured *Oligoflexaceae*
	Gamma-proteobacteria	Uncultured (*Esherichia-Shigella*)	*Rhodanobacter*
			Dokdonella
			Coxiella
			Acidiferrobacteraceae
			Ambiguous taxa (HTA4)
Chloroflexi	Ambiguous class	-	Ambiguous taxa (JG37-AG-4)
Actinobacteria	*Actinobacteria*	*Blastococcus*	*Sporichthyaceae*
		Geodermatophilus	Uncultured *Micromonosporaceae*
		Arthrobacter	
		Micrococcus	
		Agrococcus	
		Rhodococcus	
		Kocuria	
		Uncultured *Frankiales*	
	Thermoleophilia		*Solirubrobacterales* (0319-6M6)
Acidobacteria		-	-
Bacteroidetes	*Flavobacteriia*	*Chryseobacterium*	
		Flavobacterium	
	Cytophagia	*Rufibacter*	*Sporocytophaga*
		Hymenobacter	
	Sphingobacteriia	*Pedobacter*	*Sphingobacterium*
Verrucomicrobia	Ambiguous class	-	Ambiguous taxa (OPB35 soil group)
Gemmatimonadetes	*Gemmatimonadetes*	-	Gemmatimonadaceae (Uncultured soil bacterium)

			Gemmatirosa
Firmicutes	*Clostridia*	-	*Anaerococcus*
			Clostridium
	Bacilli		*Paenibacillus*
			Streptococcus

Table S2. The number of bacteria grown on agar nutrient media (thousand CFU in 1 g of soil)

Sample	T	Media	Succession points, number of days							
			**0**	**3**	**7**	**14**	**30**	**60**	**90**	**120**
BB 63-48	5°C	TSA	0	0	0	0	0	0	0	0
		R-2A	0	0	0	0	0	0	0	0
	20°C	TSA	0	19	10	0	13	35	14	4
		R-2A	0	15	0	0	16	29	3	8
BB 63-58 (AT)	5°C	TSA	15	55	220	100	179	100	287	950
		R-2A	78	130	280	190	184	350	480	670
	20°C	TSA	15	130	180	210	340	80	240	670
		R-2A	78	100	450	320	530	135	570	930
BB 63-58 (AB)	5°C	TSA	40	10	36	10	88	17	18	8
		R-2A	35	15	36	180	222	8	29	6
	20°C	TSA	40	80	1090	840	1050	1000	570	200
		R-2A	35	520	820	1325	900	1500	610	300

Table 3. Taxonomic structure of the heterotrophic bacterial complex along the course of succession

Succession points	Sample	5°C TSA	5°C R-2A	20°C TSA	20°C R-2A
0	BB 63-48	-	-	-	-
	BB 63-58 (AT)	*Variovorax ginsengisoli*	*Variovorax ginsengisoli*	-	*Cellulomonas sp.*<br>*Sphingomonas sp.*<br>*Mesorhizobium australicum*<br>*Blastococcus aggregatus*
	BB 63-58 (AB)	*Pseudomonas sp.*	*Pseudomonas sp.*	-	*Arthrobacter sp.*
3 days	BB 63-48	-	-	*Chryseobacterium montanum*<br>*Micrococcus endophyticus*<br>*Gordonia didemni*<br>*Gordonia hongkongensis/terrae*<br>*Dietzia maris*<br>*Methylorubrum podarium*	*Rhodococcus sp.*
	BB 63-58 (AT)	-	*Variovorax ginsengisoli*	-	*Variovorax ginsengisoli*
	BB 63-58 (AB)	*Pseudomonas sp.*	*Pseudomonas sp.*	*Pseudomonas sp.*	*Sphingomonas sp.*<br>*Micrococcus sp.*<br>*Arthrobacter sp.*<br>*Pseudomonas sp.*
7 days	BB 63-48	-	-	*Arthrobacter sp.*	-
	BB 63-58 (AT)	*Pseudomonas sp.*	*Pseudomonas sp.*	*Pseudomonas sp.*	*Brevundimonas vesicularis*
	BB 63-58 (AB)	*Pseudomonas sp.*	*Pseudomonas sp.*	*Bacillus halosaccharovorans*<br>*Pseudomonas sp.*	*Pseudomonas sp.*
14 days	BB 63-48	-	-	-	-
	BB 63-58 (AT)	*Pseudomonas sp.*	*Pseudomonas sp.*	*Pseudomonas sp.*<br>*Pseudoarthrobacter sp*	*Pseudomonas sp.*<br>*Mesorhizobium australicum*
	BB 63-58 (AB)	*Pseudomonas sp.*	*Pseudomonas sp.*	*Pseudomonas sp.*<br>*Pseudoarthrobacter sp*	*Pseudomonas sp.*
30 days	BB 63-48	-	-	*Methylorubrum extorquens*<br>*Arthrobacter sp.*<br>*Pseudomonas sp.*	*Methylorubrum extorquens*<br>*Arthrobacter sp.*<br>*Moraxella osloensis*<br>*Sphingomonas sp.*
	BB 63-58 (AT)	*Pseudomonas sp.*	*Pseudomonas sp.*	*Pseudomonas sp.*	*Pseudomonas sp.*<br>*Rhizobacter profundi*<br>*Mesorhizobium australicum*<br>*Bacillus sp.*

	BB 63-58 (AB)	*Pseudomonas sp.* *Agreia sp.*	*Leifsonia sp.* *Pseudomonas sp.*	*Pseudomonas sp.*	*Pseudomonas sp.* *Pseudarthrobacter sp.*
60 days	BB 63-48	-	-	*Pseudomonas sp.* *Brevundimonas vesicularis*	*Pseudomonas sp.* *Arthrobacter oryzae* *Sphingobium xenophagum* *Sphingomonas sp.* *Roseomonas sp.*
	BB 63-58 (AT)	*Pseudomonas sp.*	*Pseudomonas sp.*	*Pseudomonas sp.*	*Pseudomonas sp.*
	BB 63-58 (AB)	*Pseudomonas sp.*	*Pseudomonas sp.* *Blastococcus aggregatus*	*Pseudomonas sp.*	*Pseudomonas sp.*
90 days	BB 63-48	-	-	*Pseudomonas sp.* *Methylorubrum extorquens*	*Pseudomonas sp.*
	BB 63-58 (AT)	*Pseudomonas sp.*	*Pseudomonas sp.*	*Pseudomonas sp.* *Bacillus sp.*	*Pseudomonas sp.* *Mesorhizobium australicum*
	BB 63-58 (AB)	*Pseudomonas sp.* *Arthrobacter sp.*	*Pseudomonas sp.* *Variovorax boronicumulans*	*Pseudomonas sp.*	*Pseudomonas sp.*
120 days	BB 63-48	-	-	*Pseudomonas sp.*	*Pseudomonas sp.* *Sphingobium xenophagum* *Variovorax ginsengisoli* *Brevundimonas vesicularis* *Roseomonas sp.*
	BB 63-58 (AT)	*Pseudomonas sp.*	*Pseudomonas sp.*	*Pseudomonas sp.*	*Pseudomonas sp.* *Mesorhizobium australicum*
	BB 63-58 (AB)	*Pseudomonas sp.*	*Pseudomonas sp.* *Rhodopseudomonas sp.*	*Pseudomonas sp.* *Arthrobacter oryzae*	*Pseudomonas sp.*

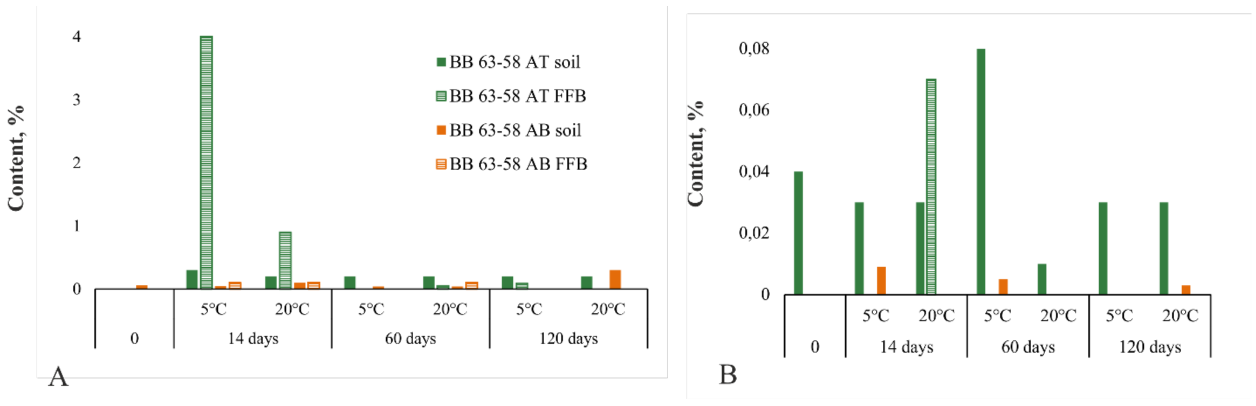

Figure 3. Content (%) of (A) phylum *Saccharibacteria*; (B) genus *Opitutus*
